# Regulatory mechanisms of Robo4 and their effects on angiogenesis

**DOI:** 10.1042/BSR20190513

**Published:** 2019-07-10

**Authors:** Chang Dai, Qiaoyun Gong, Yan Cheng, Guanfang Su

**Affiliations:** 1 Department of Ophthalmology, The Second Hospital of Jilin University, #218 Ziqiang Street, Changchun, Jilin, 130041, China; 2 Department of Ophthalmology, Shanghai General Hospital, #100 Haining Road, Shanghai, 200080, China

**Keywords:** angiogenesis, Roundabout 4, shear stress, Slit, therapy, transcription factor

## Abstract

Roundabout4 (Robo4) is a transmembrane receptor that belongs to the Roundabout (Robo) family of axon guidance molecules. Robo4 is an endothelial-specific receptor that participates in endothelial cell migration, proliferation, and angiogenesis and the maintenance of vasculature homeostasis. The purpose of this review is to summarize and analyze three main mechanisms related to the expression and function of Robo4 during developmental and pathological angiogenesis. In this review, static shear stress and the binding of transcription factors such as E26 transformation-specific variant 2 (ETV2) and Slit3 induce Robo4 expression and activate Robo4 during tissue and organ development. Robo4 interacts with Slit2 or UNC5B to maintain vascular integrity, while a disturbed flow and the expression of transcription factors in inflammatory or neoplastic environments alter Robo4 expression levels, although these changes have uncertain functions. Based on the mechanisms described above, we discuss the aberrant expression of Robo4 in angiogenesis-related diseases and propose antiangiogenic therapies targeting the Robo4 signaling pathway for the treatment of ocular neovascularization lesions and tumors. Finally, although many problems related to Robo4 signaling pathways remain to be resolved, Robo4 is a promising and potentially valuable therapeutic target for treating pathological angiogenesis and developmental defects in angiogenesis.

## Roundabout4: an endothelial-specific member of the Robo family

Roundabout (Robo) receptors, including Robo1–4, are single-pass transmembrane proteins with no autocatalytic or enzymatic activity that depend on downstream signaling and scaffolding molecules, such as the cytoplasmic kinase Abelson and the scaffolding proteins Dock/Nck, to mediate their functions [1–3]. Robo1–3 are composed of an extracellular domain with five immunoglobulin-like (Ig 1–5) domains, three fibronectin type III (Fn III 1–3) repeats, a transmembrane domain and a large, 457-amino acid cytoplasmic domain [4]. However, the molecular structure of the extracellular domain of human Robo4 only comprises two Fn repeats and two Ig domains that show the greatest homology to the first two Ig domains in human Robo1 [5,6]. Unlike other members expressed in neurons of the central nervous system (CNS), Robo4 is believed to be specifically expressed on endothelial cells (ECs) [5–7], indicating that Robo4 may participate in regulating the pathological and physiological behaviors of ECs. The stalk cell-centric Robo4 transcription in the neonatal mouse retina vascular bed suggested that Robo4 had a biological function in maintaining the differentiated and stabilized phenotype of endothelial stalk cells [8]. Robo4 knockdown or overexpression in human umbilical vein ECs (HUVECs) significantly delayed wound closure and resulted in the formation of irregular tube networks *in vitro*, indicating that a lack of and excess Robo4 expression exerted the same adverse effects on cell migration and tube formation [9]. According to Huang et al. [10], Robo4 silencing attenuated the proliferation, spreading and migration of choroid-retinal ECs (RF/6A cells).

In contrast, Cai et al. [11] discovered that the inhibition of Robo4 expression in human brain microvascular ECs (HBMECs) cultured with glioma-conditioned medium promoted the proliferation, migration and tube formation capability of ECs. Thus, the function of Robo4 depends on the context and cell/tissue type, but the specific mechanisms remain unknown. Ultimately, three main regulatory mechanisms of Robo4 have been identified: promoter control, ligand control and mechanical strain control. In this review, we will discuss each regulatory mechanism and its effect on ECs.

## Ligand regulatory mechanism

### Slits: the main ligands for Robo4

#### Slit2

The Slit proteins are secreted glycoproteins that function as repellent guidance signals for commissural axons [12,13] and include three family members termed Slit1, Slit2 and Slit3. All of these proteins share a similar domain structure, including a series of four leucine rich repeats (LRRs), seven to nine epidermal growth factor (EGF)-like domains, a laminin G domain, and a C-terminal cysteine-rich domain [7]. Slit-Robo binding has been observed for all Robo proteins, except Robo4. *Drosophila* Robo1–3 bind to a common site on the concave face of the second LRR domain of recombinant *Drosophila* Slit proteins [14]. Furthermore, human Slit2 binds to the first two Ig domains of human Robo1 or Robo2 through the second LRR sequence [15]. Based on accumulating evidence, Slit-Robo signaling requires heparan sulfate (HS) [16–19]. Two separate regions of *Drosophila* Slit, including the first two LRR domains and the C-terminal cystine knot domain, are identified as HS/heparin binding sites, while *Drosophila* Robo Ig1–Ig2 binds to heparin quite avidly [20]. However, the structure of the ternary Slit-Robo-HS signaling complex remains unknown. Thus, these findings raise the question of whether Slits bind to Robo4 and how the proteins interact.

**Figure 1:**
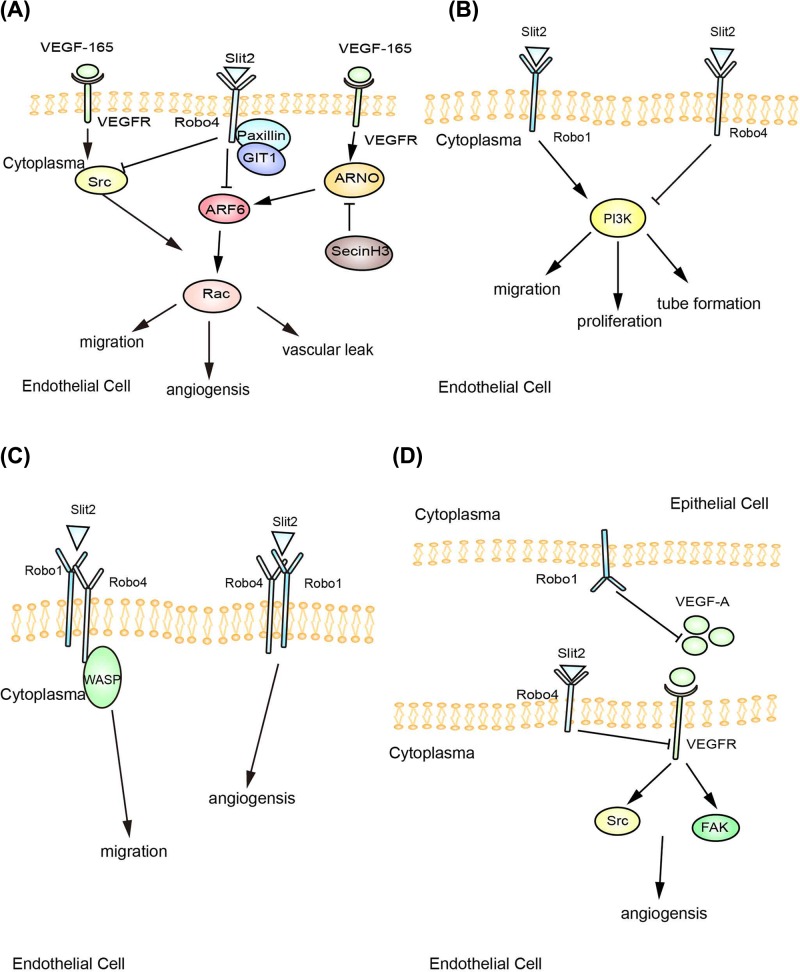
Proposed models of Slit2/Robo4 signaling in EC migration [8,9,11,25,29–31,33] (**A**) Slit2/Robo4 signaling inactivates Arf6 and inhibits SFK phosphorylation to antagonize VEGF-induced angiogenesis. (**B**) Robo1 and Robo4 competitively binds to Slit2 to affect the downstream PI3K/AKT pathway. (**C**) Slit2 binds to the Robo1-Robo4 heterodimer to modulate angiogenesis. (**D**) Robo4 transduces inhibitory Slit2 signals in the vasculature, while Robo1 inhibits proangiogenic cues under conditions favoring angiogenesis, tissue hyperplasia or pregnancy.

Slit2/Robo4 signaling has been shown to regulate cell migration [21], cell proliferation [22], and vascular permeability [23]. However, researchers disagree about the mechanism underlying the Slit2/Robo4 interaction. Some researchers believe that the activation of Robo4 alone is sufficient to inhibit EC migration in response to various promigratory factors [24–26]. Park et al. [24] observed the coimmunoprecipitation of Slit2 with Robo4, and Slit2 bound to Robo4 on intact cell membranes, supporting the hypothesis that Slit2 is the ligand for Robo4. The Slit2/Robo4 interaction inhibited cellular migration in a heterologous expression system, and the role of Robo4 in inhibiting migration was mediated by the conserved cytoplasmic motifs, namely, CC0 and CC2, of the activated receptor, which was further confirmed by the coimmunoprecipitation of the endogenous Mena protein with Robo4 in lysates from chimeric Robo4-hemaglutinin (HA)-expressing human embryonic kidney (HEK) cells. Jones et al. [25] further confirmed that the paxillin-G protein-coupled receptor kinase 2 interacting protein-1 (GIT1) complex was recruited to the cytoplasmic domain of Robo4 upon Slit2 binding to Robo4, followed by the inactivation of the small GTPase Arf6 that could activate the Rac protein responsible for protrusive activity in ECs (Figure 1A). Moreover, SecinH3, a small-molecule inhibitor of cytohesin Arf–guanine nucleotide-exchange factors (GEFs) such as Arf nucleotide binding site opener (ARNO), prevented both vascular endothelial growth factor (VEGF)-induced Arf6 activation and VEGF-induced EC migration, suggesting that Arf6 represented a critical nexus in the Slit2/Robo4 signaling pathway regulating pathological angiogenesis and vascular leakage stimulated by VEGF-165. Slit2 also inhibited the VEGF-165-dependent proliferation, migration and tube formation of ECs from the lungs of Robo4^+/+^ mice, while the inhibitory effect was abolished in Robo4^AP/AP^ ECs. These processes were mediated by reduced VEGF-165-stimulated phosphorylation of the Src family of nonreceptor tyrosine kinases (SFKs) and subsequent activation of Rac1 [8] (Figure 1A).Recently, the expression of Slit2 and Robo4, but not of Robo1, was reported to be increased in forearm skin biopsies from patients with systemic sclerosis, and the Slit2/Robo4 axis interfered with angiogenesis by inhibiting Src kinase phosphorylation [26].

However, Robo4 did not exert a critical effect on the binding site in the second LRR domain of human Slit2 (Slit2 D2) [27] and did not bind to the heparin column [28], which strengthened the Slit-Robo interaction. Thus, different models of signaling by the Slit2/Robo1–Robo4 complex have been proposed.

In the first model (Figure 1B), Slit2 directly binds to Robo1 and promotes EC migration, while Robo4 may modulate this process. As shown in the study by Enomoto et al. [29], Robo4 knockdown HUVECs showed a dose-dependent increase in migration in response to the N-terminal portion of Slit2 (Slit2N), while Robo1 knockdown or Robo1/Robo4 knockdown cells did not react to Slit2N. In addition, an incubation with soluble Robo1 (sRobo1), but not soluble Robo4 (sRobo4), blocked the Slit2N-induced migration of HUVECs [29]. The promigratory signal from Slit2 appears to be transduced through Robo1, and Robo4 may negatively modulate this process. Wang et al. [30] discovered that Slit2/Robo1 signaling increased micro-vessel densities and the tumor volumes and masses *in vivo*, and Slit2-induced directional migration and tube formation of HUVECs were attenuated by phosphatidylinositol 3-kinase (PI-3K) inhibitors *in vitro*. In another study, Cai et al. [11] discovered that Slit2/Robo4 inhibited glioma-induced EC proliferation, migration and tube formation, and that ECs of the Robo4 (-) + protein kinase B (AKT) inhibitor LY294002 group cultured with glioma-conditioned medium showed decreased cell viability, cell proliferation and tube formation compared with cells in the Robo4 (-) group. Based on these data, we propose a model in which Robo1 and Robo4 competitively bind to Slit2 to affect the downstream PI3K/AKT pathway. For the second model (Figure 1C), because Robo4 may not efficiently bind to Slit2, some researchers have proposed that Robo1 and Robo4 are coreceptors for Slit2 [9,31,32]. Increased migration of HUVECs induced by Slit2-N was suppressed by a preincubation of Slit2-N with the soluble extracellular domain of Robo1 (Robo1Fc), but not the soluble extracellular domain of Robo4 (Robo4Fc), suggesting that EC migration was mediated by Slit2 binding to Robo1, but not Robo4 [9]. Because endogenous Robo1 coimmunoprecipitated with Robo4 in HUVECs, Slit2 might still mediate its effects through Robo4, which interacted with Wiskott–Aldrich syndrome proteins (WASP) to regulate the actin cytoskeleton in ECs, despite the lack of a direct interaction. In contrast, in another study, silencing of Robo1 or Robo4 in HUVECs inhibited Slit2-mediated tube formation, and their combined loss exacerbated this effect, indicating that both Robo1 and Robo4 were cognate receptors for Slit2 that mediate angiogenesis [31]. The coimmunoprecipitation of Robo1, Robo4 and Slit2 provided direct evidence that Slit2 interacted with Robo1-Robo4 to regulate angiogenesis and maintain vasculature integrity in the placenta [31]. However, the downstream signaling pathway of Slit2/Robo1-Robo4 is unknown. For the last model (Figure 1D), Marlow et al. [33] argued that epithelial Robo1 might play a role in preventing a proangiogenic environment in addition to serving a coreceptor for Slit2. When intact Robo1 and Robo4 were simultaneously deleted in adult mice, the vasculature density and complexity increased twofold compared with wild-type (WT) mice, similar to the increase observed in Slit2^+/−^; Slit3^−/−^ mammary glands. Because the loss of Robo1 in the mammary gland, either alone or in combination with Robo4, up-regulated the expression of proangiogenic factors such as stromal derived factor-1 (SDF-1) and VEGF-A in the epithelium, the researchers further constructed chimeric mammary glands and showed that the epithelial loss of Robo1 combined with the endothelial loss of Robo4 increased the blood vessel density. Thus, endothelial Robo1 is unable to compensate for the loss of Robo4 in the endothelium, indicating that endothelial Robo4 is the main antiangiogenic receptor of Slit2, while epithelial Robo1 maintains a stable environment by negatively regulating SDF-1 and VEGF-A levels. Moreover, the activation of VEGF receptor-2 (VEGFR-2) increased in extracts from Robo1^−/−^; Robo4^−/−^ glands compared with WT, Robo1^−/−^ or Robo4^−/−^ glands. Similar to these results, VEGFR2 activation was increased in extracts from the glands of pregnant Robo4^−/−^ mice compared with pregnant WT mice, which further confirmed that loss of Robo4 under conditions favoring angiogenesis, tissue hyperplasia or pregnancy leads to increased VEGF/VEGFR2 signaling and angiogenesis.

According to the data described above, four models describe the interaction of Slit2 and Robo4. (1) Slit2 directly binds to Robo4 to inhibit angiogenesis. (2) Robo4 inhibits the Slit2-Robo1 promigratory signal. (3) Slit2 binds to the Robo1–Robo4 heterodimer to modulate angiogenesis. (4) Robo4 plays an important role in transducing inhibitory Slit2 signals in the vasculature, while Robo1 inhibits proangiogenic cues under conditions favoring angiogenesis, tissue hyperplasia or pregnancy.

#### Slit3

The chemorepellent Slit3, which is related to the migration of multiple cell types [34–37], has been recently shown to be a novel potent proangiogenic factor whose effects are mediated by Robo4 [38]. Although coimmunoprecipitation of Slit3-Robo1-Robo4 was detected, an incubation with an anti-Robo4 antibody, but not an anti-Robo1 antibody, completely inhibited the migration of HUVECs induced by Slit3 [38], indicating that the proangiogenic signal from Slit3 was transduced through Robo4. The so-called “pericytic” mesenchymal stem cells (MSCs) secreted Slit3 to direct Robo4-positive ECs to form vascular networks in engineered tissues [39]. When Zhang et al. [40] studied the etiology of congenital diaphragmatic hernia (CDH), N-deacetylase-N-sulfotransferase-1^ECKO^ (Ndst1^ECKO^) mice showed similar vascularization defects in the diaphragm to Slit3^−/−^ mice and showed more severe defects in angiogenesis when the Robo4 gene was knocked out simultaneously. Ndst1 participates in the biosynthesis of HS, which binds to Slit3 [40,41]. Based on these observations, HS may interact with Slit3/Robo4 signaling, and this pathway may underlie the etiology of CDH in Ndst1^ECKO^ mice. *In vitro*, the Slit3-induced cell migration and activation of the Rho GTPase family members were blocked in the absence of Ndst1, implying that HS functioned to facilitate Slit3-Robo4 signaling in an EC-autonomous manner. A subsequent surface plasmon resonance (SPR) spectroscopy analysis showed that Slit3 bound HS with moderate affinity, while Robo4 did not bind HS. Based on the aforementioned data, Zhang et al. [40] proposed a model in which endothelial HS presented Slit3 to Robo4 or induced a conformational change in Slit3 to the active form and the ternary complex facilitated proangiogenic signaling during developmental angiogenesis. However, Jones et al. [8] found that Slit3 blocked VEGF-165-induced Evans Blue extravasation from the retinas of Robo4^+/+^ mice, but not Robo4^AP/AP^ mice, by inhibiting VEGF-165-dependent SFK activation. Thus, it comes to a contradictory conclusion that Slit3 also activates Robo4 to suppress VEGF-165 induced vascular leakage. Slit3 and Robo4 levels were increased in preeclamptic placental tissues compared with normal controls, and hypoxia increased the expression of Slit3 and Robo4 in HUVECs [42]. Hypoxia inducible factor-1α (HIF-1α) is activated under hypoxic conditions and promotes transcription by interacting with hypoxia response elements (HREs) in the promoter regions of target genes such as VEGF and PDGF-B. As shown in the study by Fang et al. [43], HIF-1α knockdown significantly decreased Slit3 expression under hypoxic conditions, and chromatin immunoprecipitation (ChIP) assays showed stronger binding of HIF-1α to the Slit3 promoter under hypoxic conditions. Therefore, study of the HIF-1α/Slit3/Robo4 axis and its interaction with VEGF signaling in pathological neovascularization may provide new directions for anti-angiogenesis therapy.

### UNC5B

The uncoordinated-phenotype-5 (UNC5) receptor family, including UNC5A-D, was initially identified as a family of transmembrane receptors involved in axon guidance [44]. In addition to axon repulsion in the nervous system [45], these proteins also play critical roles in cell apoptosis [46], developmental angiogenesis [47] and tumor angiogenesis [48]. UNC5B is expressed in ECs *in vitro* and *in vivo* [45,49], indicating that it may interact with Robo4.

**Figure 2:**
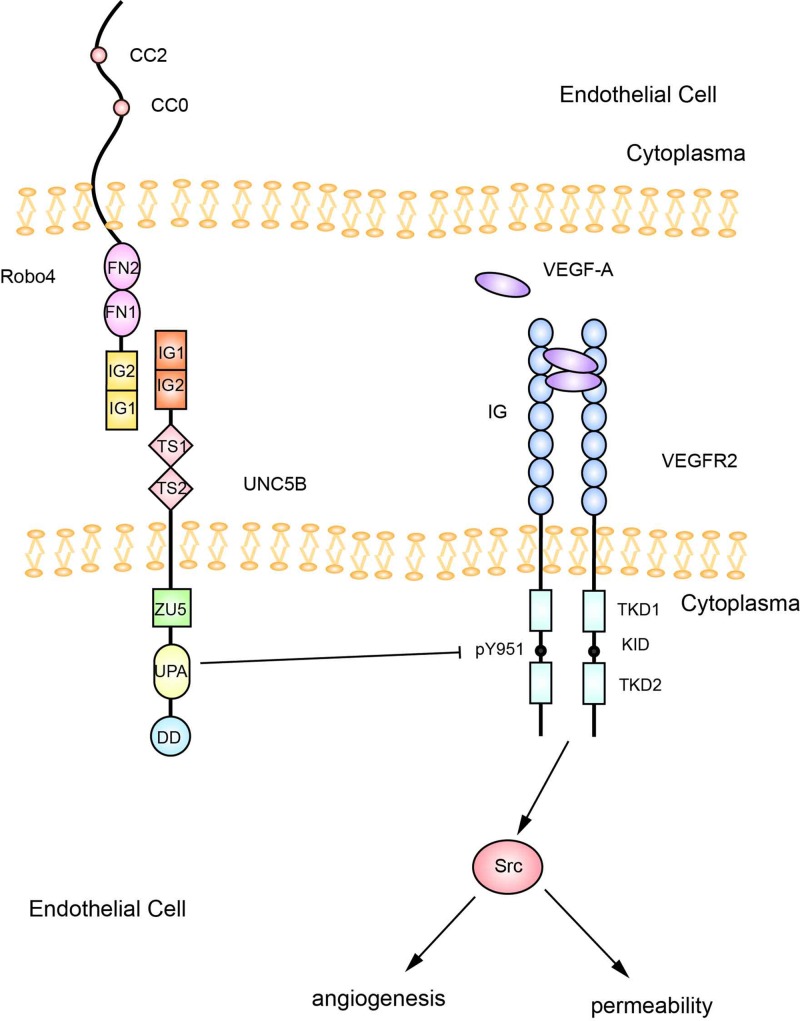
Proposed model of Robo4/UNC5B signaling [49,50]

UNC5B was initially identified as a putative Robo4 ligand through high-throughput screening of a secreted protein library [49]. Then, SPR direct binding experiments [49] further verified that Robo4-Fc specifically binds to UNC5B-Fc in a dose-dependent manner with a Kd of 12 nM. Furthermore, Robo4 was confirmed to interact with UNC5B *in vitro* and *in vivo* [49]. However, researchers have not clearly determined whether Robo4 functions as a receptor. Video microscopy [49] showed that cells expressing Robo4 lacking the cytoplasmic domain (Robo4△CD) repelled UNC5B-expressing cells, while cells expressing UNC5B lacking the cytoplasmic domain (UNC5B△CD) did not retract upon contacting 293 cells expressing full-length Robo4, indicating that the cytoplasmic signaling domain of UNC5B was necessary for repulsion. In other words, Robo4 functions as a ligand for UNC5B. Recently, researchers have discovered how the Robo4/UNC5B interaction affects the VEGF signaling pathway. After stimulating HUVECs with sRobo4, UNC5B coimmunoprecipitated with Src [49]. Furthermore, the treatment of porcine aortic ECs (PAECs) with the Src family kinase inhibitor PP2 efficiently blocked Src phosphorylation and prevented UNC5B-expressing cells from segregating from Robo4-expressing cells. When HUVECs were simultaneously incubated with VEGF and an anti-UNC5B-2 antibody, VEGF-mediated Src and phospho-Src association to VEGFR2 increased compared with the ECs treated with VEGF alone. In summary, the Robo4-UNC5B signaling pathway competes with VEGF for Src to inhibit the VEGF pathway. Zhang et al. [50] further discovered that in HUVECs, the cytoplasmic domain conserved in UNC5B, Pidd and Ankyrin (UPA domain) of UNC5B was necessary for rescuing VEGF-mediated VEGFR2 pY951 phosphorylation, which increased the levels of phosphorylated cell-sarcoma gene (c-Src) (Figure 2). Treatment with an anti-UNC5B antibody or a combination of anti-Robo4-1 and anti-UNC5B-2 antibodies exerted a similar stimulatory effect on VEGF-mediated angiogenic sprouting, supporting the hypothesis that Robo4 interacted with UNC5B to inhibit angiogenesis *in vivo* [49]. Robo4/UNC5B signaling also inhibits vascular permeability *in vivo*. Mice injected with anti-Robo4 and anti-UNC5B antibodies showed greater microsphere extravasation from retinal vessels than control mice. A Miles assay was subsequently performed to quantify vessel barrier dysfunction and showed that an intradermal injection of anti-Robo4 or anti-UNC5B antibodies mimicked the increase in basal endothelial permeability observed in Robo4^−/−^ mice. In conclusion, the Robo4/UNC5B interaction promotes the stability and integrity of vessels.

Robo4 functions as a ligand for UNC5B, and the cytoplasmic UPA domain of UNC5B reduces VEGF-mediated VEGFR2 pY951 phosphorylation to inhibit c-Src phosphorylation in HUVECs.

## Transcriptional factors and epigenetic modification

**Figure 3:**
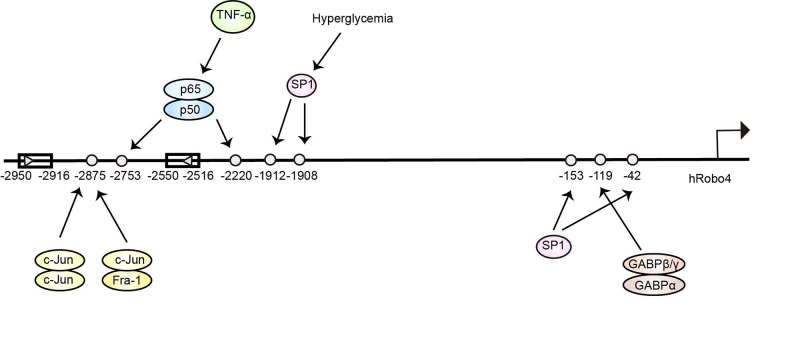
Major transcription factors regulate Robo4 expression in EC differentiation and pathological angiogenesis [51–54,58,59]

The mechanisms regulating the expression of the Robo4 gene primarily involve transcription factor-mediated control (Figure 3) and epigenetic modifications, with the former changing the promoter activity [51–53] and the latter determining the EC-specific expression [54–57]. The 3-kb upstream region of the human Robo4 promoter contains information for EC-specific expression [51]. The proximal promoter of Robo4, to which GA-binding protein (GABP) and specificity protein 1 (SP1) bind, determines the basal promoter activity. Under pathological conditions, some static transcription factor-binding motifs are activated [58,59], inducing the abnormal expression of Robo4. Epigenetic control of gene transcription includes DNA methylation, histone modification, and chromatin remodeling. DNA methylation refers to the addition of a methyl group to the 5′-position of cytosine to create 5-methyl-cytosine, which occurs almost exclusively in the context of CpG sequences in the vertebrate genome. As a repressive mark of transcriptional silencing, DNA methylation results in inhibition of Robo4 expression in non-ECs during cell differentiation. Overall, both mechanisms contribute to the differential expression of Robo4 in multiple cell lineages and various environments.

GABP and SP1 bind to the Robo4 proximal promoter to determine the basal promoter activity. The activating protein 1 (AP-1) element at position −2875 significantly enhances Robo4 promoter activity. The SP1 element at the −1912/−1908 position and the nuclear factor kappaB (NF-kB) element at the −2753/−2220 position are activated in response to hyperglycemia and inflammation, respectively, to increase Robo4 expression.

### E26 transformation-specific transcription factors

The E26 transformation-specific (ETS) family is one of the largest transcription factor families and includes 27 ETS genes in humans, 26 in mice, 10 in *Caenorhabditis elegans* and 9 in *Drosophila*. The genes are classified into 11 subfamilies (ETS, ERG, ELG, ELF, ESE, ERF, TEL, PEA3, SPI, TCF and PDEF) [60–62]. ETS proteins are characterized by their conserved winged helix-turn-helix DNA binding domain (DBD), which binds to a 5′-GGA(A/T)-3′ DNA core motif [63]. All ETS proteins, except GABPα, bind to DNA as a monomer and are auto-inhibited by two inhibitory regions that flank the DBD [62]. Furthermore, ETS proteins are involved in multiple biological processes, such as cell proliferation [64], apoptosis [65] and differentiation [66], during embryonic development and oncogenesis.

#### GABP

GABP, also known as nuclear respiratory factor 2 (NRF-2), is unique among ETS transcription factors because it is composed of GABPα and GABPβ, with the former binding to cognate DNA elements and the latter activating transcription [67]. GABP is widely expressed in various tissues and targets lineage-restricted genes, particularly myeloid genes [68–70]. Okada et al. [51] identified one ETS consensus motif at position −119 in the 3-kb Robo4 promoter that was functionally important in ECs and further assessed its binding to the transcription factor GABP through electrophoretic mobility shift assays (Figure 3). Researchers introduced a mutation into the GABP-binding motif at position −119 of the endogenous mouse Robo4 locus and observed significantly reduced activity of the mutant promoter in embryoid bodies, embryos and adult animals, supporting the hypothesis that the GABP-binding motif is essential for Robo4 expression in the intact endothelium [52]. However, in contrast to the common view that GABP activates the Robo4 promoter [52,54–56], Robo4 and VEGF expression decreased simultaneously in human Chang conjunctival epithelial cells after transfection with GABPα/β genes [71], suggesting that GABP functioned as a transcriptional suppressor of Robo4 expression in human Chang conjunctival epithelial cells. *In vivo*, similar to the conjunctival epithelial cells, Robo4 and VEGF expression decreased in the mouse cornea after the subconjunctival injection of the GABPα/β gene, decreasing the neovascularized area of the cornea [71]. These findings may be explained by the differences in the effect of GABP on the Robo4 promoter in different cell types and the regional variation in various tissues, particularly avascular regions, consistent with the hypothesis that the role of Robo4 in angiogenesis relies on the context.

#### E26 transformation-specific variant 2

**Figure 4:**
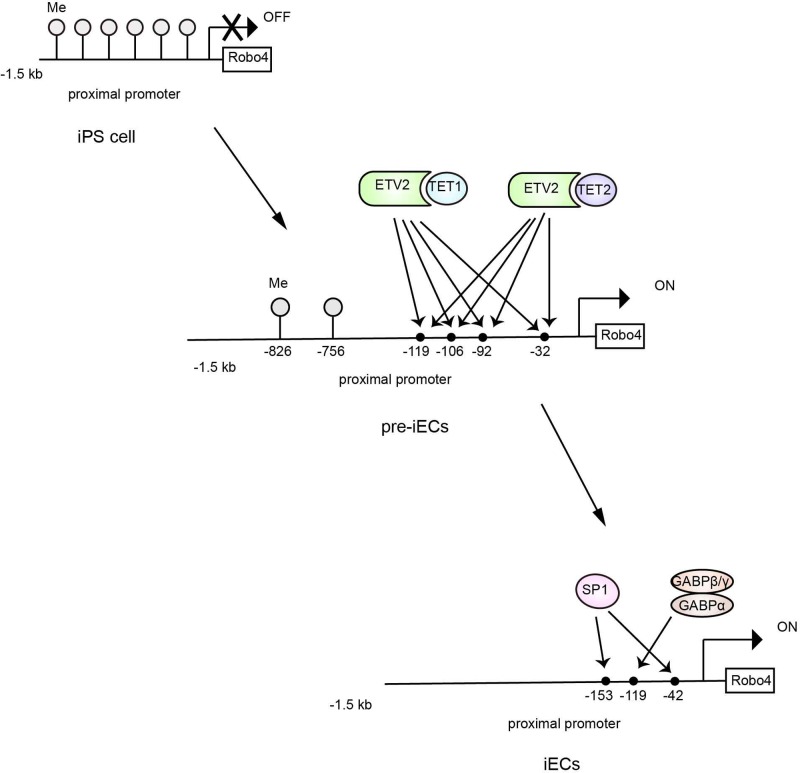
The demethylation of the proximal promoter of Robo4 during EC differentiation [57] The Robo4 promoter is highly methylated in iPS cells. When iPS cells differentiate into pre-iECs, ETV2 is expressed and recruits TET1/TET2 to the four ETS motifs to induce Robo4 expression. As cells further differentiate into ECs, ETV2 expression decreases and GABPα is recruited to the ETS(1) motif to sustain Robo4 expression.

E26 transformation-specific variant 2 (ETV2), another member of the ETS family, has attracted increasing attention due to its roles in vasculogenesis and hematopoiesis [72–75], and the protein has been recently reported to be involved in the mechanism regulating the methylation pattern of the Robo4 promoter in ECs [57]. The methylation pattern of the 300-bp proximal promoter of Robo4 differed between ECs and non-ECs [55,56]. The methylation motifs in the Robo4 promoter changed from approximately the whole promoter to a specific region between positions −2906 and −2735, with increasing expression of endothelial markers such as CD-31, Robo4 and VE-Cadherin as human induced pluripotent stem (iPS) cells differentiated into ECs [57]. Furthermore, co-transfection of ETV2, the expression of which peaked in pre-mature ECs (pre-iECs), with a reporter construct driven by the Robo4 promoter into HEK293 cells significantly increased promoter activity because ETV2 bond to four motifs in the Robo4 promoter, namely, ETS(1), ETS(2), ETS(3) and ETS(4) at positions −119, −106, −92 and −32, respectively (Figure 4). Based on these observations, ETV2 may be involved in regulating the demethylation of the Robo4 promoter when iPS cells differentiate into pre-iECs. The ten-eleven translocation (TET) demethylases TET1/TET2 were expressed at high levels during the differentiation of iPS cells and coprecipitated with ETV2, confirming this hypothesis (Figure 4). Moreover, neither TET1 nor TET2 alone could demethylate the Robo4 promoter in human dermal fibroblasts, while ETV2 could demethylate this promoter, indicating that TET1/TET2 was recruited to the Robo4 promoter via ETV2. The combination of ETV2-TET1/TET2 synergistically demethylates the Robo4 promoter and increases Robo4 promoter activity during EC differentiation (Figure 4).

Another transcription factor that may also be involved in the demethylation of the Robo4 promoter is AP-1, which binds to position −2875 followed by the binding of c-Jun and Fos-related antigen 1 (Fra-1) to this protein [53]. Because the highly methylated Region-3 containing the AP-1 motif in undifferentiated embryonic stem (ES) cells is demethylated upon the differentiation of mesodermal cells to ECs, AP-1 may be regulated by DNA methylation and involved in activating the Robo4 promoter during EC differentiation.

### The SP1 transcription factor

SP1, a eukaryotic specific factor, is involved in regulating the expression of essential oncogenes, tumor suppressors and genes related to particular cellular functions, such as matrix metalloproteinases, E-cadherin and integrin α5 [76]. Two SP1 consensus motifs are located at positions −42 and −153 of the Robo4 promoter, and the SP1 protein bind to these motifs (Figure 3) [51,54]. Mutations in the two SP1 binding sites decrease the activity of the Robo4 promoter [54]. An approximately 50% reduction in endogenous Robo4 expression was observed in primary human ECs transfected with a small interfering RNA against SP1 [51]. Recently, the SP1 mRNA was shown to be expressed at high levels in the epiretinal membranes of subjects with proliferative diabetic retinopathy (PDR), and the SP1 protein colocalized with VEGF [77], suggesting that SP1 may be involved in the mechanism regulating Robo4 expression under hyperglycemic conditions. In human retinal ECs (HRECs) transfected with SP1 siRNA and cultured under hyperglycemic conditions, the level of the Robo4 mRNA was significantly decreased, similar to the level in the normal glucose control group [59]. Moreover, transfection with a Robo4 siRNA decreased Robo4 expression less than transfection of the SP1 siRNA, and SP1 expression was not affected. Based on these results, SP1 unidirectionally regulates Robo4 expression at the transcriptional level. Subsequent ChIP assays showed that the additional SP1 binding site at positions −1912/−1908 was activated under hyperglycemic conditions to increase Robo4 expression (Figure 3) [59]. Inhibition of the SP1/Robo4 pathway by siRNAs prevents retinal vascular endothelial permeability and angiogenesis induced by a high-glucose environment and thus may serve as a therapeutic strategy for diabetic retinopathy (DR) in the future.

### The NF-kB family

The NF-kB family was discovered in 1986 as nuclear factors that bound to the enhancer element of the Ig kappa light chain of activated B cells [78]. Five family members have been identified in mammals, namely, RelA (p65), RelB, c-Rel, NF-κB1 (p50 and its precursor p105) and NF-κB2 (p52 and its precursor p100) [79]. All five family members form homo- or heterodimers through phosphorylation and other post-transcriptional modifications to enhance their DNA binding and transcriptional activities [80]. The classic NF-kB complex is the p65-p50 heterodimer, which binds to the sequence 5′-GGGRNNYYCC-3′ [81]. In most quiescent cells, these dimers are retained in the cytoplasm by binding to inhibitory molecules of the IκB family of proteins (inhibitors of NF-κB) [82]. Activation of NF-kB in inflammatory cells in response to infectious agents, inflammatory cytokines and necrotic cell products leads to the release of inflammatory, angiogenic, growth and survival factors in tumor tissues [83]. Tumor necrosis factor α (TNFα) increases the migration and incorporation of endothelial progenitor cells (EPCs) into vessel-like structures through NF-kB-mediated up-regulation of CADM1, indicating that NF-kB might modulate the functions of ECs by regulating the expression of related genes under inflammatory conditions [84]. According to Tanaka et al. [58], TNFα increased Robo4 expression in mice and human primary ECs by activating NF-kB. Mutation of the NF-kB site located at either position −2753 or −2220 attenuated TNFα-mediated promoter activation, and electrophoretic mobility shift assays revealed that the p65–p50 dimer bond to the two sites (Figure 3). The authors further observed a significant increase in the binding of both p65 and p50 to the NF-κB motifs in HUVECs stimulated with TNFα. In conclusion, high concentrations of inflammatory cytokines such as TNFα may induce Robo4 expression in ECs located in sites of inflammation and tumor vessels. The Robo4-TNF receptor-associated factor 7 (TRAF7) complex suppresses TNFα-induced hyperpermeability by stabilizing VE-cadherin at EC junctions [85]. However, further investigations are needed to determine whether the increased Robo4 expression observed under inflammatory conditions affects the proliferation, migration and high permeability of ECs.

## Shear stress control

Vascular ECs are constantly exposed to fluid shear stress generated by different blood flow patterns, which include laminar flows, oscillatory flows and disturbed flows [86,87]. Shear stress is a biomechanical force determined by the blood flow, vessel geometry and fluid viscosity. Shear stress forces are directly imposed on the endothelia and modulate the endothelial structure and function through mechanotransduction mediated by multiple mechanosensors [88–91]. Such processes result in activating shear stress response promoter elements [92,93], DNA methylation [94] and microRNAs [95,96], which modulate the expression of endothelial genes. Physiological laminar shear stress inhibits proinflammatory molecules [97], promotes vascular smooth muscle cell (VSMC) contractility [98,99] and maintains vascular integrity and homeostasis [100,101]. In contrast, oscillatory shear stress and disturbed flows activate inflammatory pathways [102], promote VSMC migration [103], and increase EC proliferation and permeability [88,104]. Robo4 is abundantly expressed in ECs from lung, liver, kidney and metastatic tumor vasculature, which is characterized by a poor blood perfusion [105]. A microarray analysis of gene expression in HUVECs revealed a marked up-regulation of Robo4 expression in the absence of shear stress [106]. Real-time PCR analyses further confirmed the predicted down-regulation of Robo4 expression in HUVECs exposed to 2 Pa of shear stress for 24 h [106], which in turn indicated that a loss of shear stress induced Robo4 expression. However, the treatment of embryoid bodies with a 10% static mechanical strain significantly increased the expression of angiogenesis-related guidance molecules, including Robo4 and ephrin B2. These changes were not reversed upon the chelation of intracellular [Ca2^+^] [107]. The down-regulation of Robo4 expression with siRNAs abolished the increase in vascular branch formation of ES cells under mechanical strain [107]. In conclusion, the angiogenic guidance molecule Robo4 participates in physiological and pathological angiogenesis modulated by differential shear stress.

## The function of Robo4 in angiogenesis

Angiogenesis is described as the formation of new blood vessels from existing vessels, which occurs both in embryonic vascular development and pathological states, such as inflammation and tumor environments. Angiogenic processes include proteolysis of the extracellular matrix and EC proliferation, migration, differentiation and tube formation. Robo4 expression was initially detected in the vasculature at sites of active angiogenesis, including the placenta and various tumors [5]. Many subsequent studies have investigated Robo4 expression in physiological and pathological angiogenesis and revealed that Robo4 is involved in regulating angiogenesis during vascular development [31,49,108] in tumors [11,109–111] and during ocular neovascularization [10,25,50,112,113].

### Robo4 and vascular development

Either Robo4 gene knockdown or overexpression destroyed the coordinated symmetric and directed sprouting of intersomitic vessels (ISV) in embryonic zebrafish [108]. Supplementation with hRobo4 compensated for ISV defects induced by Robo4 knockdown, indicating that Robo4 was essential for vascular development in vertebrates. Human placental multipotent mesenchymal stromal cell (hPMSC)-derived Slit2 and its receptor Robo4 were confirmed to regulate EC migration and tube formation to modulate angiogenesis in the placenta [31]. Robo4 was further discovered to be a novel endothelial factor contributing to the etiology of bicuspid aortic valve, a common congenital heart defect, and a reduction in the expression of or structural disruption of Robo4 in EC lines resulted in a synthetic repertoire suggestive of the endothelial-to-mesenchymal transition [114]. During retinal development, Robo4 was expressed in retinal vessels, with its level peaking at postnatal days 3 and 5, suggesting that Robo4 played important roles in stabilizing the already developed retinal vasculature instead of promoting embryonic retinal angiogenesis. This discovery was consistent with the observation that Robo4 knockout mice still showed a normal pattern of vascular development during early embryogenesis [8,115]. Recently, UNC5B has been reported to be an alternative receptor that mediates retinal vascular development, while Robo4 is dispensable for this process [49]. Controversy exists regarding the potential role of Robo4 in the embryonic development of the vasculature in vertebrates. Potential explanations for the contradictory outcomes are that although the roles of Robo4 in regional angiogenic sites may differ, the protein still plays an important role during embryonic vascular development.

### Robo4 and ocular neovascularization

Corneal angiogenesis, choroidal angiogenesis and retinal angiogenesis are major causes of vision loss in patients with a variety of clinical conditions, including stromal keratitis (SK), retinopathy of prematurity (ROP) and diabetic retinopathy. The abnormal, newly formed microvasculature that is principally stimulated by VEGF results in many problems, ranging from corneal opacification to retinal increased permeability. The Slit2/Robo4 and Robo4/UNC5B signaling pathways have been reported to stabilize ocular vessels by repressing VEGF-mediated Src kinase activation [8,49]. Here, we mainly discuss the expression and roles of Robo4 in DR and corneal neovascularization (CNV).

Diabetic retinopathy is the most common microvascular complication of diabetes and is accompanied by intense hypoxia stimulation and the increased production of proangiogenic growth factors [116]. The formation of fibrovascular membranes (FVMs) is the hallmark of PDR, which causes serious vision loss in patients. Robo4 colocalized with HIF-1α and VEGF in the vessels of FVMs and its expression increased in FVMs [117,118], indicating that it might play a role in the formation of FVMs. Under hypoxic conditions, HIF-1α positively regulated the transcription of Robo4 to promote the invasion and proliferation of HRECs [117]. Under hyperglycemic conditions, increased transcription of SP1 up-regulated the levels of Robo4 to increase the migration, permeability and angiogenesis of HRECs [59]. A similar increase in VEGF and Robo4 expression in diabetic retinas suggested that Robo4 might work together with VEGF to modulate the progression of DR. MicroRNAs represent a new therapeutic target for DR treatment, as *miR-15a-5p* inhibits the increase in VEGF and Robo4 levels in diabetic rats to delay the development of DR [118].

CNV, which is characterized by the formation of blood vessels in the avascular cornea, is a major cause of vision impairment and corneal blindness. The expression of Robo4 increased and it was located in some epithelial cells and vascular ECs invading the stroma in the neovascularized cornea compared with the normal cornea [119]. Robo4 might counteract VEGF-mediated angiogenesis, as Robo4 knockout mice showed increased CNV and SK lesions after herpes simplex virus type 1 (HSV-1) infection [113]. Some animal experiments have preliminarily explored possible strategies targeting Robo4, but had limitations. Subconjunctival administration of sRobo4 constrained CNV and SK, but it failed to produce these changes unless it was immediately injected after HSV-1 infection [113]. Subconjunctival GABP gene delivery decreased the levels of Robo4 and VEGF after 1 week, accompanied by a reduction in the vascularized areas and blood vessels in the mouse cornea [71]. However, this inhibitory effect weakened after 2 weeks. Further experimental and preclinical studies are required to optimize Robo4 targeting strategies.

### Robo4 and tumors

The tumor microenvironment is hypoxic and acidic and shows reduced blood perfusion and shear stress because of poor vessel development. Hence, tumor angiogenesis is essential for tumors because it supports the growth and metastasis of cancers. Strategies targeting tumor angiogenesis represent an alternative treatment that avoids problems of cancer cell heterogeneity. Although approaches targeting the VEGF pathway are a common and effective strategy to treat various types of cancer, VEGF inhibitors have many disadvantages, including a transient effect, resistance mechanisms and organ toxicity [120–123], partially because VEGF is also a key regulator of angiogenesis in physiological processes. Therefore, new antiangiogenic strategies might exert a specific effect when combined with VEGF inhibitors. Robo4 is differentially expressed between the normal and tumor vasculature [5,124], indicating that Robo4 expression may reflect tumor angiogenesis and become a potential target for tumor therapy. Here, we review the aberrant expression of Robo4 in cancers and summarize its potential roles and corresponding targeting strategies (Table 1).

**Table 1:** Aberrant expression of Robo4 and its functional impacts in human cancers

Cancer types	Origin	Expression	Potential roles	References
Acute myeloid leukemia	Patients	No change	Microvessel density, lower WBC	[125]
	The Cancer Genome Atlas data repository	Up-regulated	Poor prognostic factor in cytogenetic poor risk groups of patients	
Acute myeloid leukemia	Patients	Up-regulated	Poor prognostic factor for DFS and OS	[111]
Bladder cancer	Patients	Positive	Longer DSS (nuclear expression)	[126]
Bladder cancer	Patients	Positive		[127]
	T24 cells	Positive		
	T24 cells tumor-bearing mice model		Little effect on the survival and growth of the transplantation tumor	
Breast cancer	Immune competent Robo4 knockout mouse model		Suppresses breast cancer growth and metastasis	[109]
Colorectal cancer	Patients	Up-regulated		[124]
Glioma	Patients	Up-regulated	MVD values, age and glioma grade, poorer OS	[128]
Glioma	HBMECs cultured in glioma conditioned medium	Down-regulated	Suppresses glioma-induced EC proliferation, migration and tube formation *in vitro*	[11]
Glioma	Patients	Up-regulated		[129]
	Glioma cocultured hCMEC/D3 cells	Up-regulated	Suppresses BTB permeability *in vitro*	
Hepatocellular carcinoma	Patients	Down-regulated	Inversely correlated with AFP expression, discriminates liver tissues in terms of their differentiation status	[130]
Non-small-cell lung cancer	Patients	Up-regulated	Increased OS	[131]
Non-small cell lung cancer	Patients	Median serum level: 0.652 [E_450nm_]	Longer OS (serum level)	[132]
Ovarian cancer	Patients	Down-regulated		[133]
Ovarian cancer	Patients	Up-regulated		[134]
	OVCAR-3 cells, SKOV-3 cells	Positive		
Pancreatic cancer	Patients	Positive		[135]
Prostate cancer	Patients	Up-regulated	Higher Gleason score and pT stage, lower biochemical recurrence	[136]
	PC3 cells	Positive	Suppresses cell proliferation and cell viability	

**Abbreviations:** AFP, alphafetoprotein; BTB, blood–tumor barrier; DFS, disease-free survival; DSS, disease-specific survival; hCMEC/D3 cells, human cerebral microvascular endothelial cell line; MVD, microvessel density; OS, overall survival; WBC, white blood cell.

Different types of glioma-conditioned medium stimulated EC proliferation, migration and tube formation and decreased Robo4 expression, and overexpression of Robo4 suppressed these effects [11]. Slit2 expression was also down-regulated in grade III–IV glioma tissues, and the viability, migration and tube formation of ECs were significantly reduced in the Robo4 overexpression group that was pretreated with exogenous Slit2. Accordingly, Robo4 inhibits glioma-induced EC proliferation, migration and tube formation by binding to its ligand Slit2, and thus, the first Robo4 targeting strategy is the injection of high doses of the recombinant Slit2 protein. Zhao et al. [109] observed a significant increase in the percentage of the branching phenotype in tumor blood vessels in endothelial Robo4-deficient mice with a reduced level of a tight junction-associated protein, zonula occludens protein-1 (ZO-1), in tumor ECs. Treatment of breast cancer cells with SecinH3, a small molecule that could enhance Robo4 signaling by deactivating Arf6 activating proteins downstream of Robo4, significantly suppressed tumor growth, inhibited lung metastasis, reduced tumor aggressiveness and rescued the loss of the ZO-1 protein in ECs without inducing weight loss in mice [109]. Therefore, the second Robo4 targeting strategy is to target Arf6 downstream of Robo4 using the small molecule drug SecinH3. However, researchers have not determined whether globally increased levels of tight junctions also affect normal endothelial functions, particularly the blood–brain barrier. Therapies targeting the tumor vasculature include both antiangiogenic treatments and treatments that damage established tumor vessels. Tumor endothelial-specific expression of Robo4 in adults reveals that this plasma membrane protein represents an antitumor target for immunotherapeutic approaches, such as vaccination. Robo4-Fc-vaccinated mice showed retarded lung carcinoma growth, increased vascular leakage and intensified inflammation compared with control Fc-immunized mice [110]. Robo4 vaccination with alum adjuvant, a mild inducer of Th2-dependent antibody responses, also reduced tumor growth via an antibody-mediated mechanism, particularly an IgG1-mediated mechanism, instead of cytotoxic T-cell responses. The sRobo4 conjugated to a carrier protein induced a rapid protective antibody response in the absence of an adjuvant, and vaccination against Robo4 did not produce observable pathology, verifying that vaccination with the extracellular domain of Robo4 represented a promising antitumor therapy with high efficiency and no adverse effects. Because Robo4 expression is restricted to sites of active angiogenesis, including blood vessels in tumors, it represents a potential marker of the tumor vasculature when conjugated with anticancer drugs. High cell-internalizing monoclonal antibodies against Robo4 and VEGFR2 have been isolated and showed higher tumor accumulation and antitumor effects as antibody–drug conjugates (ADC) [137]. As VEGFR2 expression has also been observed in normal blood vessels, particularly in the kidney and heart, the anti-VEGFR2 antibody also recognized normal vessels and induced damage in ADC forms, while the anti-Robo4 antibody did not. The fourth Robo4 targeting therapy conjugates the high cell-internalizing anti-Robo4 antibody with anticancer drugs and targets the tumor vascular with high specificity and efficiency. In summary, although a Robo4 vaccination did not affect wound healing and organ integrity in tumor-bearing mice models, clinical evidence for the ability of Robo4-targeting strategies to avoid the known adverse effects of anti-VEGF therapy, namely, hypertension and proteinuria, is unavailable. Further animal and clinical studies are needed to confirm the efficacy and safety of Robo4-targeting therapies for tumors.

In addition to serving as an antiangiogenic target in carcinoma, Robo4 also functions as a biomarker for clinical characteristics and prognosis. Patients with acute myeloid leukemia (AML) presenting high Robo4 expression have a significantly shorter disease-free survival (DFS) and overall survival (OS) than patients with lower Robo4 expression [111]. However, increased Robo4 expression correlates with an increased OS of patients with non-small-cell lung cancer [131]. Thus, Robo4 expression and its clinical importance vary among cancers, and Robo4 may be a potential marker for disease progression during treatment with anti-tumor therapies.

## Conclusions and perspectives

In conclusion, the expression and function of Robo4 are regulated by different mechanisms, including transcription factors, Slit ligands, UNC5B ligands and mechanical strain, during physiological and pathological angiogenesis. Transcription factors and static mechanical strain during EC differentiation and vascular development induce Robo4 expression to stimulate the formation of the vasculature. In contrast, transcription factors induced by inflammation and disturbed flows also induce high levels of Robo4 expression, the functions of which remain to be explored. Regarding ligand-mediated control, Slit3/Robo4 signaling pathway promotes vascularization during tissue development, while the Slit2/Robo4 and Robo4/UNC5B signaling pathways mainly maintain vascular homeostasis by inhibiting the VEGF pathway. Annexin A2 (ANXA2) is a new Robo4 ligand that modulates cerebral trans-endothelial permeability associated with Arf6 activation [138]. Because Robo4 is abundantly expressed in areas of active angiogenesis and maintains the vascular integrity, it may function as a biomarker or target for treatments for tumors [126], inflammation-related vasculopathy [139,140] and diabetes-induced neovascularization [141]. Researchers have designed therapeutic strategies targeting different components of the Slit2/Robo4 signaling pathway, including injections of the recombinant Slit2 protein, SecinH3 injections, and anti-Robo4 antibody therapy, and verified their effects by performing animal experiments. Further studies are needed to investigate the effectiveness and safety of Robo4-targeting strategies in clinical samples.

Although we analyze the diverse expression patterns and functions of Robo4 under different circumstances in this review, the questions listed below remain to be addressed. (1) What are the physiological partners of Robo4? (2) What factors influence the choice of partners for Robo4 under pathological conditions? (3) What factors regulate the homodimerization or heterodimerization of Robo1 and Robo4? (4) What is the effect of abnormal Robo4 expression mediated by transcription factors and shear stress on angiogenesis under pathological conditions? (5) Why are Robo4-targeting therapies potentially superior to existing antiangiogenic therapies for cancers, particularly VEGF inhibitors? Most studies have supported the roles of the Slit2/Robo4 axis in inflammation [142], autoimmune diseases [26] and tumors. However, controversy still exists regarding whether Robo4 relies on its cytoplasmic domain to transmit signals that inhibit vascular permeability and neovascularization. Therefore, on one hand, studies exploring new signaling molecules upstream of Robo4 might provide new insights to increase the inhibition of neovascularization. On the other hand, as Robo1 forms dimers with Robo4 to transmit Slit2 signals, the expression and interactions of Slit2, Robo1 and Robo4, as well as their effects on tumor growth and metastasis must be studied. Anti-Robo1 antibody therapy or anti-Robo4 antibody therapy or their combination will be reasonably chosen based on the expression and functions of Robo1 and Robo4 in different tumors.

## Abbreviations

ADC: antibody-drug conjugate
AP-1: activating protein 1
ARNO: Arf nucleotide binding site opener
CDH: congenital diaphragmatic hernia
ChIP: chromatin immunoprecipitation
CNV: corneal neovascularization
c-Src: cell-sarcoma gene
DBD: DNA binding domain
DR: diabetic retinopathy
EC: endothelial cell
ES cells: embryonic stem cell
ETS: E26 transformation-specific
ETV2: E26 transformation-specific variant 2
Fra-1: Fos-related antigen 1
FVM: fibrovascular membrane
GABP: GA-binding protein
GIT1: G protein-coupled receptor kinase 2 interacting protein-1
HEK: human embryonic kidney
HIF-1α: hypoxia inducible factor-1α
HREC: human retinal EC
HS: heparan sulfate
HSV-1: herpes simplex virus type 1
HUVEC: human umbilical vein EC
iPS cell: human induced pluripotent stem cell
ISV: intersomitic vessel
LRR: leucine rich repeat
Ndst1: N-deacetylase-N-sulfotransferase-1
NF-kB: nuclear factor kappaB
OS: overall survival
PDR: proliferative diabetic retinopathy
pre-iEC: pre-mature EC
Robo: Roundabout
Robo4: Roundabout4
SDF-1: stromal derived factor-1
SFK: Src family of nonreceptor tyrosine kinases
SK: stromal keratitis
Slit2N: the N-terminal portion of Slit2
SP1: specificity protein 1
SPR: surface plasmon resonance
sRobo4: soluble Robo4
TET: ten-eleven translocation
TNFα: tumor necrosis factor α
UNC5: uncoordinated-phenotype-5
UPA domain: domain conserved in UNC5B, Pidd and Ankyrin
VEGF: vascular endothelial growth factor
VEGFR: vascular endothelial growth factor receptor
VSMC: vascular smooth muscle cell
WT: wild-type
ZO-1: zonula occludens protein-1
